# Early Learning Curve in Robotic-Assisted Total Knee Arthroplasty: A Single-Center Experience

**DOI:** 10.3390/jcm13237253

**Published:** 2024-11-28

**Authors:** David Putzer, Lennart Schroeder, Georgi Wassilew, Michael Liebensteiner, Michael Nogler, Martin Thaler

**Affiliations:** 1 Experimental Orthopaedics, Department of Orthopaedic and Trauma Surgery, Medical University of Innsbruck, Anichstrasse 35, 6020 Innsbruck, Austria; michael.nogler@i-med.ac.at; 2 Department of Orthopaedics and Trauma Surgery, Musculoskeletal University Center Munich (MUM), University Hospital LMU Munich, 80336 München, Germany; lennart.schroeder@med.uni-muenchen.de; 3 Center of Orthopaedics, Trauma Surgery and Rehabilitation Medicine, University of Greifswald, 17489 Greifswald, Germany; georgi.wassilew@med.uni-greifswald.de (G.W.); martin.thaler@helios-gesundheit.de (M.T.); 4 Department of Orthopaedic and Trauma Surgery, Medical University of Innsbruck, Anichstrasse 35, 6020 Innsbruck, Austria; 5 Arthroplasty Center Munich West, Helios Klinikum, 81241 Munich, Germany

**Keywords:** robotic-assisted surgery, total knee arthroplasty, learning curve, orthopedic surgery, robotic-assisted total knee replacement

## Abstract

**Background/Objectives**: This study evaluated the learning curve for robotic-assisted total knee arthroplasty (RA TKA) performed by three experienced surgeons, focusing on procedure duration, surgeon satisfaction, and confidence. **Methods**: A prospective study was conducted with three senior arthroplasty surgeons, each performing 15 RA TKA procedures using the Triathlon Knee System with the Robotic Arm Interactive Orthopedic (RIO) System. Data on preparation, cut-to-suture, and breakdown times were collected. Surgeon anxiety levels were measured preoperatively using the STAI-6 scale, while postoperative satisfaction and confidence were assessed via a questionnaire. Statistical analysis was conducted using GraphPad Prism. **Results**: Of 50 scheduled surgeries, 45 were completed. The average cut-to-suture time was 1 h 38 min, with significant time reductions in robotic-specific steps as experience increased. Comparing the first five surgeries to the last five, the time for navigation hardware mounting, landmarks registration, femur and tibia registration, and bone preparation decreased by up to 30% (*p* < 0.001 to *p* = 0.025). General instrument preparation time decreased by 20% (*p* = 0.004). Surgeon anxiety levels dropped, indicating increased comfort with the system, while postoperative surveys showed increased satisfaction and confidence. **Conclusions**: The study demonstrated a substantial learning curve for RA TKA, with improved efficiency and surgeon confidence by the fifteenth procedure. These findings highlight the potential for streamlined workflows and guide training for new adopters of robotic knee arthroplasty.

## 1. Introduction

The advent of robotic-assisted (RA) total knee arthroplasty (TKA) has marked a significant evolution in orthopedic surgery, promising enhanced precision, consistency, and potentially improved patient outcomes. As these technologies become increasingly integrated into clinical practice, their adoption has risen exponentially, with an analysis of the Premier Healthcare Database in the United States showing a relative increase of 601.2% in the use of RA TKA from 2015 to 2020 [1].

This surge is driven by the purported clinical benefits and growing patient demand for state-of-the-art surgical interventions, industry marketing, and the associated administrative and peer pressure [2]. Consequently, orthopedic surgeons are under mounting pressure to incorporate robotic-assisted TKA into their repertoire. This pressure, coupled with the high expectations associated with these advanced systems, necessitates a steep learning curve (LC), as shown by the number of studies on various robot-assisted techniques in this regard [3,4,5,6,7].

Previous studies on learning curves associated with RA TKA indicated a longer operation time [3]. Several of these studies were conducted almost 15 years ago, revealing that robot-assisted procedures took up to 30 min longer than traditional TKA, leading to skepticism among surgeons toward these technologies [2,3]. However, recent research suggests that this concern diminishes after the learning phase, with recent studies indicating that RA TKA can be performed within a timeframe comparable to conventional TKA [3,4,8,9,10,11,12,13,14,15].

Evaluating the duration of the learning curve is crucial, as it allows for predicting future task completion time and efficiency. Such assessment may also aid in designing training programs [7] and in predicting reductions in operation time with increasing routines, tailored to the specific task requirements [3]. However, to generalize the learning curve of a new technique, it is essential to minimize potential confounding factors that could influence the analysis. These factors include variations in surgeon specialization, TKA volume, the gradual introduction of RA TKA, surgeons’ experience with RA TKA systems, and consistency of surgical settings [8,10,16,17].

The aim of this study was to evaluate the impact of individual worksteps in the early learning curve for robot-assisted TKA among three experienced arthroplasty surgeons within the same department in a standardized setting. This was achieved by observing the first 15 robot-assisted TKAs performed by each surgeon and assessing procedure duration and the surgeons’ satisfaction using a validated questionnaire.

## 2. Materials and Methods

This study was conducted as a prospective study at a single center, whereby intraoperative data was obtained from three surgeons specializing in hip and knee arthroplasty. The learning curve consists of two phases: learning phase and proficiency phase. Tay et al. concluded that in robotic-arm-assisted total knee arthroplasty, after an initial learning curve of 16 cases, surgeons enter into a consolidation phase [14]. Vermue showed an inflection of the learning curve within a range of 11 to 43 cases [16]. Jung et al. determined a learning curve of 18 cases with robotic-arm-assisted TKA [12]. Depending on the experience level, the moment surgeons reached proficiency varies considerably, indicating that the learning curve is not the same for everyone [13]. Kayeani et al. and Schopper et al. have shown that after 7–9 cases, the learning phase was changing into a proficiency phase [4,10]. To evaluate the learning experience in the early learning phase, a total of 15 robotic-arm-assisted surgeries were performed by each of the 3 Senior Endocert-certified arthroplasty surgeons (>100 arthroplasties a year) and used for the analysis [18]. In all cases, the Triathlon Knee System was implanted with a Robotic Arm Interactive Orthopedic (RIO) system (Stryker, Kalamazoo, MI, USA). All three surgeons were newly introduced to the robotic arm system and participated in a cadaver course, where the main functions of the system were taught. One surgeon had extensive experience with navigation systems. Each surgeon completed 15 TKA within 2 years (from November 2019 until October 2021). As time is the most common determinant of the learning curve, it was used as the primary measure [5]. IRB approval was not required as the study focuses primarily on the surgeon’s performance. To perform an accurate assessment, the surgical workflow was divided into steps with lengths that could be measured, including the standard and robot arm-assisted specific steps, through which the learning process of the three surgeons in our study center could be evaluated.

The work steps were evaluated by independent observers who were also asked to read the surgical manual on the instruction for use of the robotic system. Each observer participated in several standard knee surgeries and analyzed a surgical video for training purposes. A specific software was used to record the period of each step, which were as follows.

The recorded “preparation time” started with the first action of the scrub tech or the Mako Product Specialist (MPS) and ended with the time out before the incision. “Cut to suture time” started with the first incision and ended with the last stitch performed on the patient. The “breakdown time” recorded every step after the completion of wound closure until the patient left the surgical theatre.

The “preparation time” includes several steps. “Setup robotic arm” includes the assembly of the system, the draping of the robotic arm, and the mounting of the cutting tool on the robotic arm by the scrub tech as well as its registration. It ends with mounting the sawblade on the device. “General instruments setup” starts with the scrub tech unpacking the sterile disposables and ends when all necessary instruments are set on the tables and the disposables are unpacked. As the robotic device was not fully integrated into the daily routine, the scrub techs prepared both the specific robotic instruments as well as all instruments for the specific implant system (Triathlon Knee System, Stryker, Kalamazoo, MI, USA). “Patient preparation” includes the disinfection of the surgical site and patient draping as well as connecting all necessary cables and tubes; the final step was to apply the incision foil to the patient. In some surgeries, for the “mounting legholder” step, a dedicated leg positioner (Leg Positioner, Stryker, Kalamazoo, MI, USA) was used. The recording of the step started when the surgeon mounted the clamp on the table and finished when the patient’s leg was fixed in the legholder.

“Incision and joint exposure” includes all steps performed by the surgeon to expose the patient’s knee joint. “Mounting navigation hardware and landmarks registration” includes all steps necessary to assemble the tracking devices for the tibia and femur, insert the checkpoints, register them, and record the patient-specific landmarks (hip rotation center and malleoli) at the patient’s surgical site. “Femur registration” is the time required to capture 40 points on the femoral bone surface and verify the match between the virtual bone model and the real bone surface. “Tibia registration” is the time required for collecting 40 points on the tibial bone surface and the verification of the registration process. The step “Ligament balancing” involves the removal of osteophytes that restrict knee joint mobility, the recording of the joint space (closest distance between the femoral surface and deepest point on the tibia surface in the medial and lateral compartment of the knee joint) in full knee extension and 90° of knee flexion, and the adjustment of the implant position following the decision of the surgeon based on the assessed joint gaps and the relative tension of the ligaments. “Bone preparation” includes the positioning of the robotic arm in its cutting position and the sequence of the 6 bone cuts necessary to insert the knee implant. All cuts were performed in the following order: distal femur cut, posterior chamfer femur cut, changing of sawblade attachment, posterior femur cut, anterior femur cut, anterior chamfer femur cut, and tibial cut. In “Trialing” the following steps performed by the surgeon were recorded: insertion of the trial implants, assessment of the range of motion, all manual bone preparation steps (removal of cutting edges, removal of remaining osteophytes, box preparation for PS implants, drilling peg holes on the femur, keel insertion on the tibia), confirmation of final implant sizes, and removal of the trial implants. “Implantation of components” includes the steps of inserting the femoral and tibial implant on the prepared bone surfaces using bone cement, the insertion and the removal of a trial inlay, checking range of motion, inserting the final inlay and the final check of range of motion. “Waiting for cement hardening” was defined as the time when only limited surgical actions were performed and the leg was kept in full extension. “Removal of navigation hardware” includes the removal of checkpoints and array pins inserted in the patient to perform the robotic-arm-assisted procedure. “Suturing” covers the time from the first to the last stitch of the wound closure.

“Break down time” included the following steps.

The step “Patient postoperative care” includes removing all attached devices, undraping the patient, bandaging the leg, taking a postoperative X-ray in the operating room, and discharging the patient from the operating room. “Surgical instruments break down” includes all the necessary steps to remove all items from the tables of the scrub tech. “Robotic arm breakdown“ includes removing the cutting tool and array from the robotic arm as well as the undraping of the device.

A six-item state anxiety test (STAI-6) derived from the Spielberger State-Trait Anxiety Inventory (STAI) based on a 5-point Likert scale was used to assess the pre-operative questionnaire. The STAI is one of the most used validated measures of anxiety in health research [19]. The 6 items used were 1: calm, 3: tense, 6: upset, 15: relaxed, 16: content, and 17: worried, described as the highest anxiety-present and the anxiety-absent items [19,20] with a total score range from 6 to 30; a higher score indicates a higher level of anxiety. The overall score was converted into a scale of 20–80. An STAI score ≤ 35 indicates no anxiety, scores of 36–41 indicate moderate anxiety, and scores ≥ 42 indicate severe anxiety [19].

The postoperative questionnaire consisted of 10 questions for the surgeon, also based on a 5-point Likert scale, taking into account the following:

- Satisfaction: I’m very satisfied with the way the surgery went today;
- Confidence: I’m sure that the joint function has been normalized as well as possible, I’m sure that the implant has been optimally aligned, I’m sure that the tension of the ligaments has been set ideally for this patient, I’m sure that the bones were precisely prepared, I’m very happy with the tracking today;
- Working load-reduced (National Aeronautics and Space Administration Task Load Index NASA TLX questionnaire) [21,22]: I was able to operate the robotic arm without thinking, working with the robotic arm wasn’t physically stressful for me;
- Team interactions: The team communication worked very well today;
- Team familiarity: the teamwork in the work processes with the robotic arm worked very well today.

All statistical analyses and graphical representations were conducted using Graph Pad Prism 8.0.1 software (GraphPad Software, Inc., La Jolla, CA, USA). *p*-values *p* < 0.05 were considered statistically significant. All data were tested for normal distribution using the Kolmogorov–Smirnov Test (*p* < 0.05). Comparison of the duration of single work steps between surgeons was analyzed using ANOVA. To evaluate progress in reducing surgical time for each parameter assessed (preparation time, cut-to-suture time, etc.), the first five surgeries of each surgeon were compared to the 11–15th surgeries of each surgeon using the unpaired *t*-test. Answers to the questionnaires recorded at the first five surgeries of each surgeon were compared to the 11–15th surgeries and analyzed with the Mann–Whitney U test for statistical significance. The learning curve of the operative time was determined using cumulative summation (CUSUM) analysis, according to previous studies [12,16]. Logarithmic regression lines were used to evaluate time progression. Logarithmic regression was calculated for the learning curves to determine the steepness of the learning curves. Slope, *R*^2^, and 95% confidence interval were reported.

A priori power analysis for 3 groups with an effect size of 0.5 and α-error of 0.05 resulted in a sample size of 45 with an actual power of 0.905. Power analysis was performed with G*Power 3.1.9.7 (G*Power, Universität Kiel, Kiel, Germany).

## 3. Results

Fifty surgeries were scheduled from November 2018 to July 2021. One of the surgeries had to be canceled due to missing preoperative planning due to a faulty CT scan (2019), one surgery could not be performed due to technical reasons with the robotic arm, three surgeries were canceled for health issues of the patient, and three surgeries were converted into non-robotic cases during the COVID pandemic as either patient or MPS were affected by health issues. Of the remaining 45 cases, each of the three surgeons participating in the study performed 15 cases.

In 2018 the first surgery was performed, in 2019 the number of surgeries was 20, in 2020 there were 13, and in 2021, 11 surgeries were performed with the robotic arm system. Out of the 45 cases, five were planned by the MPS while the remaining preoperative plans were submitted to the company’s segmentation service. The median time between performing a CT scan and receiving the preoperative plan from the external segmentation team was 3 days, with a minimum of sending it back within the same day and a maximum duration of 59 days to a longer turnaround time.

**Figure 1:**
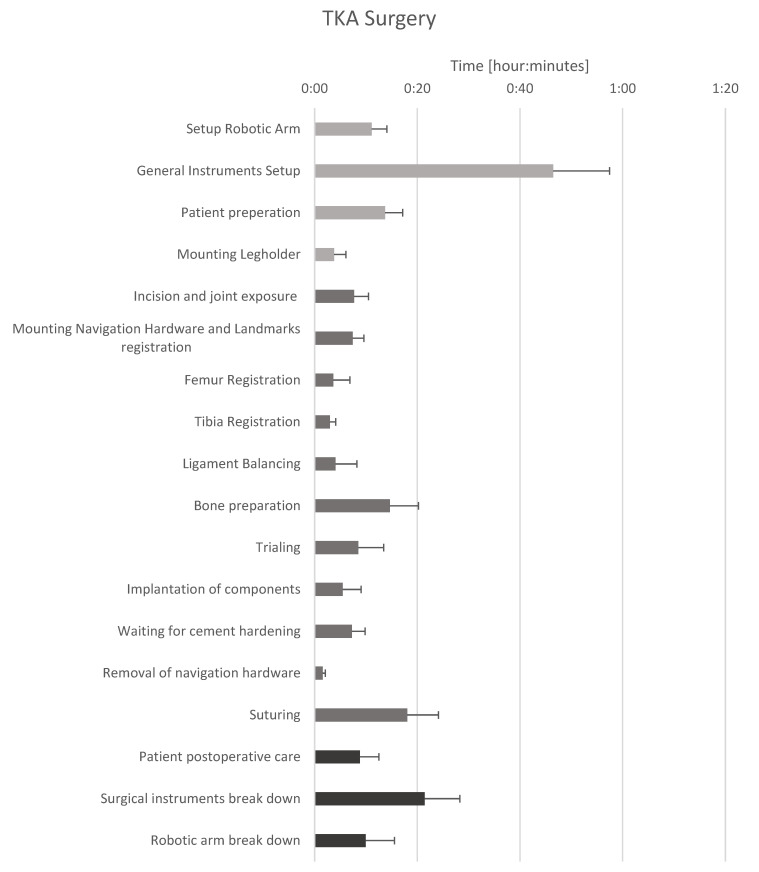
The mean duration of single work steps during robotic-assisted total knee replacement of 45 surgeries. Bars indicate the standard deviation.

The average cut-to-suture time was 1 h and 38 min (SD 23 min). The average preparation time was 1 h 18 min (SD 19 min) and the average break-up time was 42 min (SD 15 min) (Figure 1). All data were normally distributed (*p* < 0.05). No difference was found between surgeons in the time between incision and suture (*p* = 0.763), preparation time (*p* = 0.650), and breakdown time (*p* = 0.655).

The workflow was divided into steps and time recordings were performed (Table 1, Figure 1). The preparation time was divided into setting up the robotic arm, general instruments setup, patient preparation, and mounting of a dedicated legholder for robotic-arm-assisted surgery. The median time for setting up the robotic arm was 10 min (range, 6–19 min), the median time for general instruments setup, including unpacking all disposables, was 46 min (range, 19–70), while patient preparation, including patient washing and draping, lasted for 12 min (range, 8–21) on average. The legholder was only used by one surgeon in all cases, while one surgeon stopped using the device after four cases and the third surgeon used it initially for the first five cases and only used it again in the last case. The median time for setting up the device was 3 min (range, 1–10 min).

Table 1 reports a median preparation time of 81 min, a median surgery time of 98 min, and a median breakdown time of 38 min. The surgery itself was divided into 11 steps. The surgical approach accounts for up to 8% of the total operating time. Tracker assembly accounts for up to 7% of the total surgery time. The bone registration takes 2 min per bone (1–6 min for the tibia and 1–23 min for the femur), which accounts for up to 6% of the total surgery time. In one case, femoral registration was performed multiple times and took 23 min due to a moving tracker array. Ligament balancing as well as intraoperative plan adjustments took up to 2 min with a range between 0 (no balancing required for the patient) and 18 min (difficult cases which required intensive plan adjustments). Ligament balancing took up to 2% of the surgical time. The robotic bone preparation accounted for 13% of the total surgery time. The trialing and final component implantation took 11 min, 4 min (range, 2–27) for trialing and 4 min (1–21) for final implantation, that accounted for up to 13% of the total surgery time. Waiting for cement hardening accounted for up to 7% of the total surgery time. The suturing accounted for up to 18% of the total surgery time.

Patient postoperative care required a median time of 8 min, robotic arm breakdown required a median time of 8 min, and surgical instruments breakdown required 20 min.

**Figure 2:**
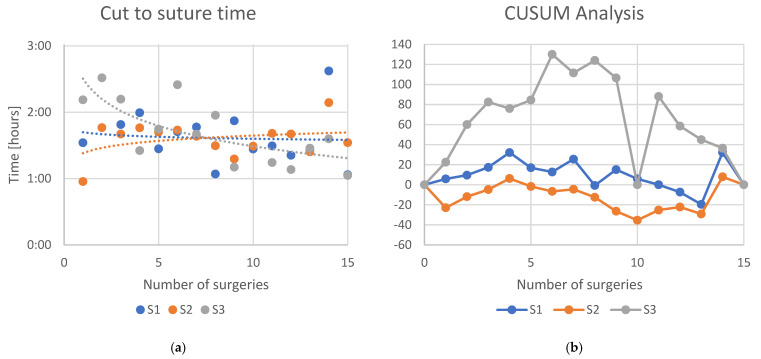
(**a**) Learning curves of the three surgeons. Dots indicate the time each surgeon needed to complete the surgery. Lines indicate an interpolated logarithmic regression line. (**b**) CUSUM analyses of the learning curves of the three surgeons for the cut-to-suture time.

No statistically significant difference could be found for the cut-to-suture time (*p* = 0.1240) for the three surgeons under evaluation. Indeed, as shown in Figure 2a, S1 and S2 showed no improvement within the surgeries and the logarithmic regression had a slope of 0.002 (*R*^2^ = 0.006) for S1 and a slope of 0.004 (*R*^2^ = 0.117) for S2. S3 showed a logarithmic regression line with slope= −0.018 (*R*^2^ = 0.526). CUSUM analysis of the total surgical time showed a deflection for S3 between the 6th and 8th case, while in S1 and S2 the learning effect was not visible (Figure 2b) for the cut-to-suture time.

To analyze the LC, the first five and the last five surgeries from each surgeon were compared (Table 2). The learning curves of mounting the legholder were not analyzed, as none of the three surgeons used the device consistently. In the dataset of the femoral registration, an outlier was removed (23 min, multiple registrations due to loose femoral array) for further analysis of the learning curves. All data was normally distributed (*p* < 0.05). A statistically significant time reduction could be found for the three surgeons, when comparing their first five surgeries to their last five surgeries, concerning mounting the navigation hardware and landmarks registration (*p* < 0.001), femur registration (*p* = 0.003), tibia registration (*p* = 0.001), and bone preparation (*p* = 0.025) (Table 2). The time saved in all four steps was up to 30%. The team reduced the preparation time of the general instruments by 20% (*p* = 0.004).

**Figure 3:**
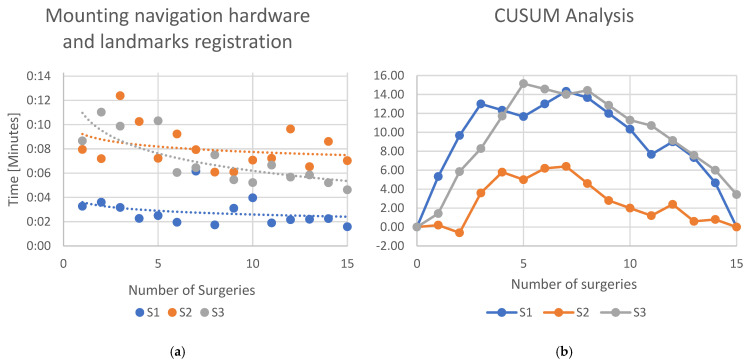
(**a**) Learning curves of the three surgeons for the specific workstep “Mounting navigation hardware and landmark registration”. Dots indicate the time each surgeon needed to complete the workstep. Lines indicate an interpolated logarithmic regression line. (**b**) CUSUM analysis of the learning curve.

The steps which showed a statistically significant difference were further analyzed, determining the logarithmic regression line and performing CUSUM analysis. Considering the robotic-specific step “Mounting navigation hardware and landmarks registration”, the logarithmic regression line showed a slope of −0.0003 (*R*^2^ = 0.087) for S1 and a slope of −0.0005 (*R*^2^ = 0.084) for S2, while S3 showed a slope of −0.001 (*R*^2^ = 0.624) (Figure 3a). The CUSUM analysis (Figure 3b) showed that for S1 and S2, the learning curve declined after the 7th case, while for S3, the learning curve declined after the 8th case.

**Figure 4:**
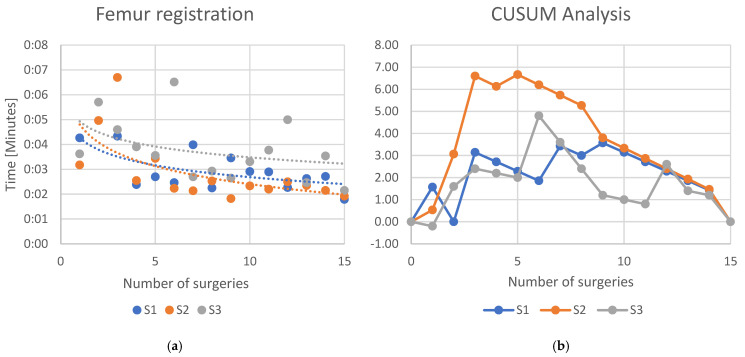
(**a**) Learning curves of the three surgeons for the specific workstep “Femur registration”. Dots indicate the time each surgeon needed to complete the workstep. Lines indicate an interpolated logarithmic regression line. (**b**) CUSUM analysis of the learning curve.

For the robotic-specific step “Femur registration”, the logarithmic regression line showed a slope of −0.0005 (*R*^2^ = 0.415) for S1, −0.0007 (*R*^2^ = 0.383) for S2, and −0.0004 (*R*^2^ = 0.159) for S3 (Figure 4a). The CUSUM analysis (Figure 4b) showed a declination of the learning curve for S1 and S2 after the 9th case, while for S3, the learning curve declined after the 5th case.

**Figure 5:**
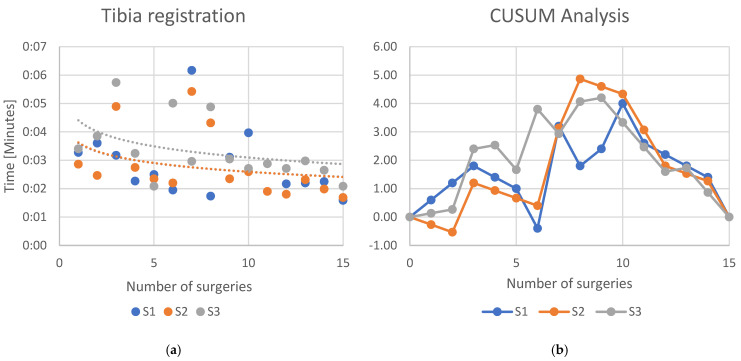
(**a**) Learning curves of the three surgeons for the specific workstep “Tibia registration”. Dots indicate the time each surgeon needed to complete the workstep. Lines indicate an interpolated logarithmic regression line. (**b**) CUSUM analysis of the learning curve.

For the robotic-specific step “Tibia registration” the logarithmic regression line with slope of −0.0003 (*R*^2^ = 0.087) was calculated for S1, a slope of −0.0003 (*R*^2^ = 0.094) was calculated for S2, while S3 showed a slope of −0.0004 (*R*^2^ = 0.171) (Figure 5a). The CUSUM analysis (Figure 5b) showed an inclination of the learning curve for S1 after the 10th case, S2 after the 8th case, while for S3 the learning curve declined after the 9th case.

**Figure 6:**
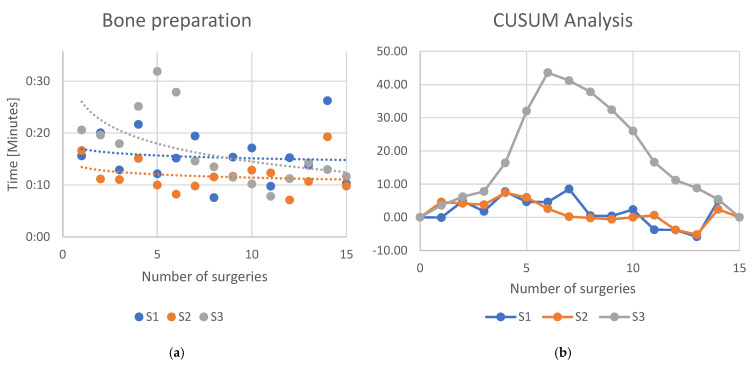
(**a**) Learning curves of the three surgeons for the specific workstep “Bone preparation”. Dots indicate the time each surgeon needed to complete the workstep. Lines indicate an interpolated logarithmic regression line. (**b**) CUSUM analysis of the learning curve.

For the robotic-specific step “bone preparation” S1 showed a logarithmic regression line with slope of −0.0006 (*R*^2^ = 0.016), S2 showed a slope of −0.0006 (*R*^2^ = 0.049), while S3 showed a slope of −0.0003 (*R*^2^ = 0.310) (Figure 6a). The CUSUM analysis (Figure 6b) showed an inclination of the learning curve for S1 after the 7th case and S2 after the 4th case, while for S3, the learning curve declined after the 6th case.

The results of the assessed questionnaires are shown in Table 3. Preoperatively, surgeons became calmer (*p* = 0.006), less tension was reported (*p* = 0.006), nervousness decreased (0.001), and surgeons became more relaxed (0.005) when comparing the answers in the questionnaires between the first five surgeries and the last five surgeries. No differences in the preoperative satisfaction (*p* = 0.868) as well as no difference of being worried about the upcoming surgery were observed (*p* = 0.099) between the initial five surgeries and the last five surgeries.

When determining the overall score and converting it into the STAI-6 score, a statistically significant reduction (*p* = 0.001) in anxiety was found between the first five surgeries and the last five surgeries. The median anxiety level was ≤35, which indicates no anxiety in most of the cases. The highest level of anxiety was reached in one case (47) at the time of the first surgery by one of the surgeons, which indicates strong anxiety before surgery. One surgeon’s anxiety level changed from moderate to anxiety-free, while the third surgeon still experienced moderate anxiety in one case during the last five surgeries performed.

Postoperatively, an increase in general satisfaction was observed (*p* = 0.037). Confidence levels increased for tracking of the patient (*p* = 0.034). The reported mental workload decreased significantly (*p* = 0.001) as well as the physical stress in using the robotic arm (*p* = 0.031). Team familiarity increased significantly (*p* = 0.001). No difference could be observed between the first five surgeries and the last five surgeries in terms of confidence about the joint function, implant position, ligament balancing, and bone preparation (*p* > 0.05). In these cases, confidence remained at the highest level and could not be further improved. The same could be observed for the team interaction, where no statistically significant difference could be found (*p* = 0.118) as all three surgeons were satisfied with the team performance in the first five surgeries as well as in the last five surgeries.

## 4. Discussion

With an increasing number of robotic-assisted TKA and a corresponding increase in surgeons’ interest in adopting these technologies, the learning curve for RA TKA is crucial to predict cost management associated with early phase adoption as well as surgeons’ perception during the learning phase.

In a study by Kayani et al. about the LC of robotic-arm-assisted TKA in 2019, they found an LC of seven cases, but this study included only one surgeon, and the surgical team was already experienced in working with navigated TKA system [4]. In another study by Sodhi et al. about the LC associated with robotic TKA in 2018, they compared two surgeons in different hospitals performing manual and robotic TKA, but they did not mention after how many cases the curve levels out [3]. Schopper et al. reported an inflection point of the learning curve on the 9th case [10]. Jung et al. reported an inflection point in the learning curve after 18 cases [12]. Ejnsiman et al. observed that surgeons who reached the proficiency stage were able to reduce robotic-assisted TKA surgical times to below those of conventional TKA after approximately 30 robotic procedures [13]. This reduction highlights the potential for robotic surgery to match or surpass conventional methods in efficiency once proficiency is achieved [13].

It is worth noting that the point at which robotic-assisted TKA surgical time equals or exceeds conventional TKA varies significantly. While Kenanidis et al. reported that 70 surgeries might be required for time equalization [23], other studies have suggested a learning curve of fewer than 25 cases [11,15]. Eniysman emphasized that the learning curve is highly individualized and influenced by factors such as surgeon skill, experience, and familiarity with the specific robotic system [13]. Surgical time determined for the three surgeons in our study is comparable to the study of Schopper et al. [10] and also comparable to other robotic surgical systems [24].

In this study, we were able to show that in 15 robotic-assisted surgeries performed by three different surgeons, the time reduction of the robotic steps was 33% on average. No difference was found in any of the steps that are also performed during a standard operation. The average cut-to-suture time was 1 h and 28 min in the first five surgeries and lowered to 1 h and 16 min for the 10th to 15th surgeries.

The median for preoperative planning was 3 days; however, this was mainly influenced by the availability of patients getting a CT scan, hospital administration, and surgical planning. With a more streamlined process for the planning procedure and the availability of CT scanners, the time between CT scans and surgery could be shortened even further. When implementing a robotic system based on images with a specific protocol, a considerable amount of time should be allowed for planning, as in some cases editing or even re-scanning of the images may be required. In our case, one faulty CT scan resulted in the cancellation of the surgery. It is therefore recommended to perform the CT scan at least one week before surgery.

On average, surgery preparation and breakdown time lasted 3 h and 38 min, not including the cleaning time between surgeries. Preparation time was 1 h and 18 min and break down time about 42 min, while the average time from incision to suture was 1 h and 38 min.

For the preparation, 57% of the time the scrub tech was busy unpacking sterile disposables and preparing the general instruments (42 min). The time for setting up the instruments might be reduced by creating specific instrument sets for robotic-assisted surgeries and by reducing the number of disposable items. However, in bigger hospitals, this might be possible only when all conventional TKAs are converted to robotic-assisted TKAs. Setting up the robotic arm was performed in 10 min, including setting up the device, performing all safety checks, calibrating the device, and draping as well as mounting all sterile components on the device itself. If a dedicated legholder was used, four additional minutes were required by the surgical team. Patient preparation required an additional 12 min and also included attaching the surgical equipment, e.g., suction station, electrocauter as well as covering the staff with sterile gowns. Some processes run in parallel, while on the other hand, the scrub tech has to wait for the sterile equipment to be handed over. The efficiency of the process is heavily influenced by the scrub tech and his team’s interactions. The impact of setting up the hardware for a robotic-assisted surgery can take up to 17% of the overall preparation time and should be taken into account for daily planning. In this case, we considered a worst-case scenario, when the robotic device has to be set up for each case. The preparation time for the robotic device might change significantly if the setup process is required only once a day, while calibration and mounting of the sterile equipment are required for each case.

When considering the cut-to-suture time, it was found that the shortest surgery lasted 57 min and the longest lasted 2 h and 37 min. Within the learning curve of 15 surgeries per surgeon, we observed a median cut-to-suture time of 1 h and 38 min. 26% of that time was mainly used for incision and joint exposure and for suturing at the end of surgery. By using more minimally invasive approaches, a significant reduction of time may be possible. Preparing the bone surface (cutting with the robotic device) took up 13% of the time. Even in this case, the effects on the duration of the entire procedure can be considerable, depending on the surface being treated (minimum 7 min and maximum 32 min). Mounting the required navigation hardware, bone, and landmark registration, as well as the removal of the navigation hardware, required 15% of the overall cut-to-suture time. Curing of the cement required 7% of the overall cut-to-suture time. By using non-cemented implants, the cut-to-suture time can be reduced by this value. Trialing and implantation of components are also required for conventional steps and accounted for 13% of the cut-to-suture time. Depending on the degree of misbalance of the knee joint (no ligament balancing required to extensive ligament balancing required), this step can take up to 18 min, especially during the learning phase where some additional explanations might be necessary. Overall, this process requires only 2% of the whole cut-to-suture time and it is well invested, as this might influence all other steps including recutting scenarios. In one case, femoral registration was performed several times due to an unstable tracking array. Breakdown time mainly depends on the removal and packaging of the surgical instruments by the scrub tech which takes up to 53% of the overall median breakdown time of 38 min. Breaking down the robotic arm requires 21% of the time and the same amount is required for bandaging the patient, performing a final X-ray in the surgical theatre, and removing the patient from the surgical theatre.

The learning curve of the three experienced surgeons and the surgical team can be observed in the following steps.

In the general instrument preparation, a significant time reduction of 20% could be achieved. Surgeons were able to reduce the time required for mounting navigation hardware and bone registration by 33%. The bone preparation procedure could be shortened by 29%. Overall, the cut-to-suture time was reduced from 1 h 21 min to 1 h 11 min, although it was not statistically significant.

All four of the robotic-specific steps, “mounting navigation hardware and bone registration”, “femoral registration”, “tibia registration”, and “bone preparation”, influenced the learning curves of the surgeons, while in all other surgical steps no statistically significant difference could be found. After the 8th case, all three surgeons gained enough proficiency for “mounting the navigation hardware and bone registration”. Femur registration took up to 9 cases and tibia registration took up to 10 cases until all three surgeons reached a declination of their learning curve in the CUSUM analysis. The learning curve of the surgeons decreased after the 7th case for the “Bone preparation”, which consisted of the robotic-arm-assisted sawing of the femur and tibia. It can be deduced that the step of “bone preparation” was surprisingly the easiest to familiarize, followed by the step “mounting navigation hardware and bone registration”. The bone registration method, which is crucial during the procedure, required a slightly longer learning curve, especially for the tibia.

Our results are well in line with the study of Kayani et al., who observed a significant reduction in surgical time after the initial seven cases of robotic-assisted total knee arthroplasty [4]. The improvement was attributed mainly to enhanced efficiency in bone registration and bone preparation as surgeons became more adept at using the robotic arm and gained better control over its movements [4].

The bone preparation time in our study was initially superior in the first five cases (17 min) than the time reported in the study of Tay et al. in the first 10 cases (13 min) [14]; however, after 15 cases (16 cases in the study of Tay et al.), the same time was achieved in both studies (8.8 min) [14]. In the study of Tay et al. a mean approach and bone registration time of 16 min was reported for the first 10 cases [14], while the surgeons in our study achieved a mean time of 23 min for the first five cases. After the first 10, the time was reduced to 17 min, while the study of Tay et al. reported 13 min [14]. However, this difference might be attributed to different start and end points of the measurements or differences in the workflow. When considering the time for joint balancing in the study of Tay et al., they improved from 8.4 min in the first 10 cases to 8 min in the following 10 cases [14]. Kayani et al. reported a mean time for joint balancing during the proficiency state of 8.9 min [4]. In our study, 5 min were used for the ligament balancing in the first five cases and 2 min for the following 10 cases.

Higher levels of anxiety and stress were observed in our study at the initial phase of the learning curve, which was also reported by Kayani et al. [4] and Londhe et al. [25].

No significant differences were observed when comparing the operation time, range of motion, and complication rates between robotic-assisted TKA and conventional TKA, although robotic-assisted TKA is capable of achieving superior alignment in several axes [26,27]. Robotic-assisted TKAs potentially offer improved health outcomes as lower annualized revision rates and enhanced postoperative quality of life can be observed, especially when annual institutional case volume is higher than 24 cases annually [28,29,30].

Limitations of the study were that the learning curves were assessed by three different surgeons in a single-center study. Measurements of the single worksteps depend on the observer taking the measurements. However, we have tried to define recognizable start and endpoints for the measurements to enable a precise measurement. Scrub techs were different in every surgery, which might be different in other hospitals but reflects a worst-case scenario. No patient data was included in this study as we wanted to concentrate on the performance of the surgeons during the single worksteps and their learning curve. Another limitation lies in the circumstances imposed by the COVID-19 pandemic, which likely affected all elective surgical disciplines. Reduced elective surgical capacity and case volume may have significantly decreased the number of procedures performed, potentially disrupting the development of the learning curve for robotic-assisted TKAs in this case series. Additionally, the absence of a control group limits the ability to precisely evaluate learning curve behavior, leaving the findings to be interpreted as trends. Establishing a true control group would be challenging, as each surgeon can only experience a single learning curve for a given technique.

## 5. Conclusions

Previous studies on the learning curve for robotic-assisted total knee arthroplasty show that a minimum of seven cases are required with an experienced team. In our study, we could also see a significant time reduction within the first 15 surgeries. The total time for surgery preparation, execution, and breakdown averaged 3 h and 38 min, excluding cleaning time. Preparation took 1 h and 18 min, breakdown 42 min, and the cut-to-suture time averaged 1 h and 38 min. Preoperative planning time was influenced by patient availability for CT scans, hospital administration, and surgical planning, averaging 3 days. A streamlined process could reduce this time, and it is recommended to perform CT scans at least one week before surgery to avoid cancellations. Significant time reductions were observed in various steps: 20% in general instrument preparation, 33% in mounting navigation hardware and bone registration, and 29% in bone preparation. The overall cut-to-suture time decreased from 1 h and 21 min to 1 h and 11 min, which represents a significant improvement for the entire team.

## Figures and Tables

**Table 1:** The median of each operative parameter analyzed, with its minimum and maximum measured in minutes.

Work Step	Median (Range) [min]	
Preparation time	81 (26–115)	% of preparation time
Setup robotic arm	10 (6–19)	13
General instruments setup	46 (19–70)	57
Patient preparation	12 (8–21)	16
Mounting legholder	3 (1–10)	4
Cut-to suture-time	98 (57–157)	% of cut-to-suture time
Incision and joint exposure	7 (2–13)	8
Mounting navigation hardware and Landmarks registration	7 (2–12)	7
Femur registration	2 (1–23)	3
Tibia registration	2 (1–6)	3
Ligament balancing	2 (0–18)	2
Bone preparation	13 (7–32)	13
Trialing	7 (2–27)	8
Implantation of components	4 (1–21)	5
Waiting for cement hardening	7 (2–16)	7
Removal of navigation hardware	1 (1–3)	2
Suturing	17 (9–33)	18
Breakdown time	38 (18–100)	% of breakdown time
Patient postoperative care	8 (3–21)	21
Surgical instruments breakdown	20 (8–41)	53
Robotic arm breakdown	8 (2–28)	21

**Table 2:** Mean, improvement, and *p*-value of the time recording of the operative parameters.

Work Step	Mean of First 5 Surgeries (SD) [min]	Mean of Last 5 Surgeries (SD) [min]	Time Reduction [%]	*p*-Value
Preparation time	81 (18)	71 (23)		0.302
Setup robotic arm	12 (4)	9 (3)		0.101
General instruments setup	55 (9)	41 (11)	20	0.004
Patient preparation	15 (4)	13 (4)		0.1667
Cut-to-suture time	88 (17)	76 (23)		0.1240
Incision and joint exposure	8 (3)	7 (3)		0.394
Mounting navigation Hardware and landmarks registration	9 (2)	6 (2)	33	<0.001
Femur registration	3 (1)	2 (1)	33	0.003
Tibia registration	3 (1)	2 (1)	33	0.001
Ligament balancing	5 (6)	2 (1)		0.1164
Bone preparation	17 (6)	12 (5)	29	0.025
Trialing	9 (4)	8 (6)		0.688
Implantation of components	5 (2)	6 (5)		0.288
Waiting for cement hardening	7 (2)	7 (3)		0.218
Removal of navigation hardware	1 (1)	1 (1)		0.776
Suturing	20 (6)	16 (5)		0.069
Break down time	41 (13)	44 (21)		0.743
Patient postoperative care	8 (3)	10 (5)		0.189
Surgical instruments break down	21 (7)	22 (7)		0.782
Robotic arm breakdown	12 (7)	9 (4)		0.209

**Table 3:** Mean, improvement, and *p*-value of the pre- and post-operative questionnaires.

	Parameter	Median of First 5 Surgeries (Range) [min]	Mean of Last 5 Surgeries (SD) [min]	*p*-Value
preoperative	Calm	5 (2–5)	5 (5–5)	0.006
	Tense	1 (1–3)	1 (1–1)	0.006
	Nervous	2 (1–5)	1 (1–1)	0.001
	Relaxed	4 (2–5)	5 (1–5)	0.005
	Satisfied	5 (3–5)	5 (1–5)	0.868
	Worried	1 (1–5)	1 (1–1)	0.099
	STAI-6	30 (20–47)	20 (20–40)	0.001
postoperative	Satisfaction after surgery	4 (1–5)	5 (2–5)	0.037
	Confidence on joint function	4 (2–5)	5 (3–5)	0.214
	Confidence on implant position	5 (2–5)	5 (3–5)	0.328
	Confidence on ligament balancing	5 (3–5)	5 (4–5)	0.257
	Confidence on bone preparation	5 (2–5)	5 (5–5)	0.099
	Confidence on tracking	4 (1–5)	5 (4–5)	0.034
	Mental working load on robotic arm usage	3 (1–5)	5 (3–5)	0.001
	Physical working load when using the of robotic arm	4 (1–5)	5 (2–5)	0.031
	Team interaction	5 (3–5)	5 (4–5)	0.118
	Team familiarity	4 (1–5)	5 (4–5)	0.001

## Data Availability

Due to privacy restrictions the data will be available on request of the corresponding author.

## References

[1] Wang J.C., Piple A.S., Hill W.J., Chen M.S., Gettleman B.S., Richardson M., Heckmann N.D., Christ A.B. (2022). Computer-Navigated and Robotic-Assisted Total Knee Arthroplasty: Increasing in Popularity Without Increasing Complications. J. Arthroplast..

[2] Sherman W.F., Wu V.J. (2020). Robotic Surgery in Total Joint Arthroplasty: A Survey of the AAHKS Membership to Understand the Utilization, Motivations, and Perceptions of Total Joint Surgeons. J. Arthroplast..

[3] Sodhi N., Khlopas A., Piuzzi N.S., Sultan A.A., Marchand R.C., Malkani A.L., Mont M.A. (2018). The Learning Curve Associated with Robotic Total Knee Arthroplasty. J. Knee Surg..

[4] Kayani B., Konan S., Huq S.S., Tahmassebi J., Haddad F.S. (2019). Robotic-Arm Assisted Total Knee Arthroplasty Has a Learning Curve of Seven Cases for Integration into the Surgical Workflow but No Learning Curve Effect for Accuracy of Implant Positioning. Knee Surg. Sports Traumatol. Arthrosc..

[5] Pernar L.I.M., Robertson F.C., Tavakkoli A., Sheu E.G., Brooks D.C., Smink D.S. (2017). An Appraisal of the Learning Curve in Robotic General Surgery. Surg. Endosc..

[6] Kayani B., Konan S., Pietrzak J.R.T., Huq S.S., Tahmassebi J., Haddad F.S. (2018). The Learning Curve Associated with Robotic-Arm Assisted Unicompartmental Knee Arthroplasty. Bone Jt. J..

[7] Mazzon G., Sridhar A., Busuttil G., Thompson J., Nathan S., Briggs T., Kelly J., Shaw G. (2017). Learning Curves for Robotic Surgery: A Review of the Recent Literature. Curr. Urol. Rep..

[8] Grau L., Lingamfelter M., Ponzio D., Post Z., Ong A., Le D., Orozco F. (2019). Robotic Arm Assisted Total Knee Arthroplasty Workflow Optimization, Operative Times and Learning Curve. Arthroplast. Today.

[9] Savov P., Tuecking L.-R., Windhagen H., Ehmig J., Ettinger M. (2021). Imageless Robotic Handpiece-Assisted Total Knee Arthroplasty: A Learning Curve Analysis of Surgical Time and Alignment Accuracy. Arch. Orthop. Trauma. Surg..

[10] Schopper C., Proier P., Luger M., Gotterbarm T., Klasan A. (2023). The Learning Curve in Robotic Assisted Knee Arthroplasty Is Flattened by the Presence of a Surgeon Experienced with Robotic Assisted Surgery. Knee Surg. Sports Traumatol. Arthrosc..

[11] Vaidya N., Gadekar A., Agrawal V.O., Jaysingani T.N. (2023). Learning Curve for Robotic Assisted Total Knee Arthroplasty: Our Experience with Imageless Hand-Held Navio System. J. Robot. Surg..

[12] Jung H.J., Kang M.W., Lee J.H., Kim J.I. (2023). Learning Curve of Robot-Assisted Total Knee Arthroplasty and Its Effects on Implant Position in Asian Patients: A Prospective Study. BMC Musculoskelet. Disord..

[13] Ejnisman L., Antonioli E., Cintra L., de Oliveira Souza P.G., Costa L.A.V., Lenza M. (2024). Robot-Assisted Knee Arthroplasty: Analyzing the Learning Curve and Initial Institutional Experience. Comput. Struct. Biotechnol. J..

[14] Tay M.L., Carter M., Zeng N., Walker M.L., Young S.W. (2022). Robotic-Arm Assisted Total Knee Arthroplasty Has a Learning Curve of 16 Cases and Increased Operative Time of 12 Min. ANZ J. Surg..

[15] Mahure S.A., Teo G.M., Kissin Y.D., Stulberg B.N., Kreuzer S., Long W.J. (2022). Learning Curve for Active Robotic Total Knee Arthroplasty. Knee Surg. Sports Traumatol. Arthrosc..

[16] Vermue H., Stroobant L., Thuysbaert G., de Taeye T., Arnout N., Victor J. (2023). The Learning Curve of Imageless Robot-Assisted Total Knee Arthroplasty with Standardised Laxity Testing Requires the Completion of Nine Cases, but Does Not Reach Time Neutrality Compared to Conventional Surgery. Int. Orthop..

[17] Meghpara M.M., Goh G.S., Magnuson J.A., Hozack W.J., Courtney P.M., Krueger C.A. (2023). The Ability of Robot-Assisted Total Knee Arthroplasty in Matching the Efficiency of Its Conventional Counterpart at an Orthopaedic Specialty Hospital. J. Arthroplast..

[18] Haas H., Grifka J., Günther K.P., Heller K.D., Niethard F.U., Windhagen H., Ebner M., Mittelmeier W. (2013). EndoCert—Zertifizierung von EndoProthetikZentren in Deutschland.

[19] Marteau T.M., Bekker H. (1992). The Development of a Six-item Short-form of the State Scale of the Spielberger State—Trait Anxiety Inventory (STAI). Br. J. Clin. Psychol..

[20] Tluczek A., Henriques J.B., Brown R.L. (2009). Support for the Reliability and Validity of a Six-Item State Anxiety Scale Derived from the State-Trait Anxiety Inventory. J. Nurs. Meas..

[21] Cao A., Chintamani K.K., Pandya A.K., Ellis R.D. (2009). NASA TLX: Software for Assessing Subjective Mental Workload. Behav. Res. Methods.

[22] Colligan L., Potts H.W.W., Finn C.T., Sinkin R.A. (2015). Cognitive Workload Changes for Nurses Transitioning from a Legacy System with Paper Documentation to a Commercial Electronic Health Record. Int. J. Med. Inform..

[23] Kenanidis E., Boutos P., Sitsiani O., Tsiridis E. (2023). The Learning Curve to ROSA: Cases Needed to Match the Surgery Time between a Robotic-Assisted and a Manual Primary Total Knee Arthroplasty. Eur. J. Orthop. Surg. Traumatol..

[24] Dragosloveanu S., Petre M.A., Capitanu B.S., Dragosloveanu C.D.M., Cergan R., Scheau C. (2023). Initial Learning Curve for Robot-Assisted Total Knee Arthroplasty in a Dedicated Orthopedics Center. J. Clin. Med..

[25] Londhe S.B., Rudraraju R.T., Shah R.V., DeSouza C., Shetty V., Khan F.S., Bajwa S. (2024). Analysis of Robot-Specific Operative Time and Surgical Team Anxiety Level and Its Effect on Alignment during Robot-Assisted TKA. J. Robot. Surg..

[26] Ren Y., Cao S., Wu J., Weng X., Feng B. (2019). Efficacy and Reliability of Active Robotic-Assisted Total Knee Arthroplasty Compared with Conventional Total Knee Arthroplasty: A Systematic Review and Meta-Analysis. Postgrad. Med. J..

[27] Onggo J.R., Onggo J.D., De Steiger R., Hau R. (2020). Robotic-Assisted Total Knee Arthroplasty Is Comparable to Conventional Total Knee Arthroplasty: A Meta-Analysis and Systematic Review. Arch. Orthop. Trauma. Surg..

[28] Rajan P.V., Khlopas A., Klika A., Molloy R., Krebs V., Piuzzi N.S. (2022). The Cost-Effectiveness of Robotic-Assisted Versus Manual Total Knee Arthroplasty: A Markov Model–Based Evaluation. JAAOS-J. Am. Acad. Orthop. Surg..

[29] Hua Y., Salcedo J. (2022). Cost-Effectiveness Analysis of Robotic-Arm Assisted Total Knee Arthroplasty. PLoS ONE.

[30] Vermue H., Tack P., Gryson T., Victor J. (2021). Can Robot-Assisted Total Knee Arthroplasty Be a Cost-Effective Procedure? A Markov Decision Analysis. Knee.

